# Fractional modeling dynamics of HIV and CD4^+ ^T-cells during primary infection

**DOI:** 10.1186/1753-4631-6-1

**Published:** 2012-01-03

**Authors:** AAM Arafa, SZ Rida, M Khalil

**Affiliations:** 1 Department of mathematics, Faculty of Science, South Valley University, Qena, Egypt; 2 Department of mathematics, Faculty of Engineering, Modern Science and Arts University (MSA), Giza, Egypt

## Abstract

In this paper, we introduce fractional-order into a model of HIV-1 infection of CD4^+ ^T cells. We study the effect of the changing the average number of viral particles *N *with different sets of initial conditions on the dynamics of the presented model. Generalized Euler method (GEM) will be used to find a numerical solution of the HIV-1 infection fractional order model.

## 1. Introduction

At the present time there are several countries, particularly in Africa, with up to 35% of their populations between the ages of 15 and 50 years infected by human immunodeficiency virus (HIV) [1]. Throughout the world, already over 16 million deaths have been caused by this virus. HIV is a retrovirus that targets the CD4^+ ^T lymphocytes, which are the most abundant white blood cells of the immune system. Although HIV infects other cells also, it wreaks the most havoc on the CD4^+ ^T cells by causing their decline and destruction, thus decreasing the resistance of the immune system [2,3]. Mathematical models have been proven valuable in understanding the dynamics of HIV infection [4-6]. In 1989, Perelson developed a simple model for the primary infection with HIV [7]. This model has been important in the field of mathematical modeling of HIV infection, and many other models have been proposed, which take this model as their inspiration. Perelson et al. extended the model in 1993 and discussed some of the model's behavior [8]. They defined the model by considering four categories: uninfected CD4^+ ^T cells, latently infected CD4^+ ^T cells, productively infected CD4^+ ^T cells and virus population.

We will consider some models for HIV-1 population dynamics below [9]. Here there are two components: *x*, the number of uninfected CD4^+ ^T -cells and y, the number of infected such cells. Then the following two equations describe the evolution of the system:

$$\begin{array}{ccc} \frac{dx}{dt} & =s-\mu x-\beta xy & \\ \frac{dy}{dt} & =\beta xy-\nu y \end{array}$$

where all parameters and variables are non-negative. *s *is the assumed constant rate of production of CD4^+ ^T -cells, *μ *is their per capita death rate, *βxy *is the rate of infection of CD4^+ ^T -cells by virus, and *vy *is the rate of disappearance of infected cells. The viral variable has been omitted for simplicity as it is here assumed to be linearly related to *y*. A more complete model of human immunodeficiency virus type 1 (HIV-1) dynamics considers in addition to the uninfected and infected CD4^+ ^T -cells, *x *and y respectively, the number of virions in plasma, *z*. The following three equations are:

$$\begin{array}{ccc} \frac{dx}{dt} & =s-\mu x-\beta xz, & \\ \frac{dy}{dt} & =\beta xz-\nu y,\\ \frac{dz}{dt} & =cy-\gamma z-\beta xz. \end{array}$$

The third equation in the last mentioned reference does not contain the term *-βxz *to account for the fact that when a virus infects a CD4^+ ^T -cell, *z *decreases at the same time as *x *decreases. Without this we have:

$$\begin{array}{ccc} \frac{dx}{dt} & =s-\mu x-\beta xz, & \\ \frac{dy}{dt} & =\beta xz-\nu y,\\ \frac{dz}{dt} & =cy-\gamma z. \end{array}$$

Rong et al. further modified the model by incorporating anti-retroviral effects to study the evolution of drug resistance [10]. They considered three classes of CD4^+ ^T cells: uninfected cells, infected cells in eclipse phase and productively infected cells. The model depends on the observation that for a virus, when it enters a resting CD4^+ ^T cell, viral RNA may not be completely reverse transcribed into DNA. In [11], the authors modified the ODE model proposed by Culshaw and Ruan into a system of fractional-order [12]. They showed that the model established in this paper possesses non-negative solutions, as desired in any population dynamics. They obtained a restriction on the number of viral particles released per infectious cell, in order for infection to be sustained. Following Rong et al., we assume here that a fraction of infected CD4^+ ^T-cells return to the uninfected class. In view of this, the following model is proposed:

(1) $$\begin{array}{ccc} \frac{dT}{dt} & =s-KVT-dT+bI, & \\ \frac{dI}{dt} & =KVT-\left(b+\delta \right)I,\\ \frac{dV}{dt} & =N\delta I-cV. \end{array}$$

With initial conditions

$$T\left(0\right)=T_{0},I\left(0\right)=I_{0},V\left(0\right)=V_{0}.$$

In this model, *T, I *and *V *denote the concentration of uninfected CD4^+ ^T cells, infected CD4^+ ^T cells, and free HIV virus particles in the blood, respectively. *δ *represents death rate of infected T cells and includes the possibility of death by bursting of infected T cells, hence *δ *≥ *d*. The parameter *b *is the rate at which infected cells return to uninfected class while *c *is death rate of virus and *N *is the average number of viral particles produced by an infected cell. The rest of the paper is organized as follows. Section 2 gives an idea about fractional calculus. In section 3, we introduce fractional-order into the model that describes HIV infection of CD4^+ ^T cells. Section 4 gives an idea about the generalized Taylor's formula while section 5 presents the idea of generalized Euler's method for solving FODEs. Section 6 is devoted for the numerical results.

## 2. Fractional calculus

Fractional calculus has been extensively applied in many fields [13,14]. Many mathematicians and applied researchers have tried to model real processes using the fractional calculus. Jesus, Machado and Cunha analyzed the fractional order dynamics in botanical electrical impedances [15,16]. Petrovic, Spasic and Atanackovic developed a fractional-order mathematical model of a human root dentin. In biology, it has been deduced that the membranes of cells of biological organism have fractional-order electrical conductance [17] and then are classified in groups of non-integer order models. Fractional derivatives embody essential features of cell rheological behavior and have enjoyed greatest success in the field of rheology [18]. Fractional order ordinary differential equations are naturally related to systems with memory which exists in most biological systems. Also, they are closely related to fractals, which are abundant in biological systems. Hence, we propose a system of FODE for modeling HIV. We first give the definition of fractional-order integration and fractional-order differentiation [19]. There are several approaches to the generalization of the notion of differentiation to fractional orders e.g. Riemann-Liouville, Caputo and Generalized Functions approach. For the concept of fractional derivative, we will adopt Caputo's definition, which is a modification of the Riemann-Liouville definition and has the advantage of dealing properly with initial value problems.

**Definition 1**. The fractional integral of order *α *> 0 of a function *f*: *R*^+^→ *R *is given by

(2) $$J^{\alpha }f\left(x\right)=\frac{1}{\Gamma \left(\alpha \right)}\overset{x}{\underset{0}{\int }}{\left(x-t\right)}^{\alpha -1}f\left(t\right)dt,$$

Where *J*^0^*f(x) *= *f(x), α *> 0, *x *> 0.

**Definition 2**. Riemann-Liouville and Caputo fractional derivatives of order *α *where *α *∈ (*n*-1, *n*) of a continuous function *f*: *R*^+^→ *R *is given respectively by

(3) $$D^{\alpha }f\left(x\right)=D^{m}\left(J^{m-\alpha }f\left(x\right)\right),$$

(4) $$D_{*}^{\alpha }f\left(x\right)=J^{m-\alpha }\left(D^{m}f\left(x\right)\right),$$

Where *m*-1 <*α *≤ *m, m *∈ *N*.

The reason of using fractional order differential equations is that they are naturally related to systems with memory which exists in most biological systems. Also they are closely related to fractals which are abundant in biological systems. The definition of fractional derivative involves an integration which is non local operator (as it is defined on an interval) so fractional derivative is a non local operator. In other word, calculating time fractional derivative of a function *f *(*t*) at some time *t *= *t*_1 _requires all the previous history, i.e. all *f *(*t*) from *t *= 0 to *t *= *t*_1 _The results derived of the fractional systems are of a more general nature. However, the fundamental solutions of these equations still exhibit useful scaling properties that make them attractive for applications. We would like to put your attention that time fractional derivatives change also the solutions we usually get in standard system. The concept of fractional or non-integer order derivation and integration can be traced back to the genesis of integer order calculus itself. Most of the mathematical theory applicable to the study of non-integer order calculus was developed through the end of 19^th ^century. However it is in the past hundred years that the most intriguing leaps in engineering and scientific application have been found. The calculation technique has in some cases had to change to meet the requirement of physical reality. The derivatives are understood in the Caputo sense. The general response expression contains a parameter describing the order of the fractional derivative that can be varied to obtain various responses. One of the basic reasons of using fractional order differential equations is that ***"Fractional order differential equations are, at least, as stable as their integer order counterpart."***

## 3. Fractional-order model derivation

Now we introduce fractional-order into the model (1) of HIV infection of the CD4^+ ^T -cells. The new system is described by the following set of FODEs of order *α*_1_, *α*_1_, *α*_3 _> 0:

(5) $$\begin{array}{ccc} D^{\alpha_{1}}\left(T\right) & =s-KVT-dT+bI, & \\ D^{\alpha_{2}}\left(I\right) & =KVT-\left(b+\delta \right)I,\\ D^{\alpha_{3}}\left(V\right) & =N\delta I-cV. \end{array}$$

## 4. Generalized Taylor's formula

In this section we introduce a generalization of Taylor's formula that involves Caputo fractional derivatives. This generalization is presented in [20].

Suppose that

$D_{*}^{k\alpha }f\left(x\right)\in C\left(0,a\right],for\;k=0,1,...,n+1,$ where 0 <*α *≤ 1. Then we have

(6) $$f\left(x\right)=\overset{n}{\underset{i=0}{\sum }}\frac{x^{i\alpha }}{\Gamma \left(i\alpha +1\right)}\left(D_{*}^{i\alpha }\right)\left(0+\right)+\frac{\left(D_{*}^{\left(n+1\right)\alpha }f\right)\left(\xi \right)}{\Gamma \left(\left(n+1\right)\alpha +1\right)}x^{\left(n+1\right)\alpha }$$

With 0 ≤ *ξ *≤ *x*, ∀ *x *∈ (0, *a*].

In case of *α *= 1, the generalized Taylor's formula (6) reduces to the classical Taylor's formula.

## 5. Generalized Euler method (GEM)

Most nonlinear fractional differential equations do not have analytic solutions, so approximations and numerical techniques must be used [21]. The decomposition method (ADM) and the variational iteration method (VIM) are relatively new approaches to provide an analytical approximate solution to linear and nonlinear problems, and they are particularly valuable as tools for scientists and applied mathematicians, because they provide immediate and visible symbolic terms of analytic solutions, as well as numerical approximate solutions to both linear and nonlinear differential equations. In recent years, the application of the ADM, VIM, in linear and nonlinear problems has been developed. On the other hand, these methods are effective for small time, i.e. *t *< < 1, however the standard homotopy perturbation method (HPM) cannot solve the problem for larger time and in fact the solution of the chaotic system using HPM is an open problem. Nevertheless by chance, there are cases at which these methods give good approximation for a large range of time (*t*). A few numerical methods for fractional differential equations have been presented in the literature. However many of these methods are used for very specific types of differential equations, often just linear equations or even smaller classes. Odibat and Momani derived the generalized Euler's method that we have developed for the numerical solution of initial value problems with Caputo derivatives [22]. The method is a generalization of the classical Euler's method. Consider the initial value problem

(7) $$D_{*}^{\alpha }y\left(t\right)=f\left(t,y\left(t\right)\right),y\left(0\right)=y_{0},$$

For 0 <*α *≤ 1, t > 0.

Let [0, *a*] be the interval over which we want to find the solution of the problem (7). In actuality, we will not find a function *y*(*t*) that satisfies the initial value problem (7). Instead, a set of points {*t*_*j*_, *y*(*t*_*j*_)} is generated, and the points are used for our approximation. For convenience we subdivide the interval [0, *a*] into *k *subintervals [*t*_*j*_, *t*_*j *+ 1_] of equal width *h *= *a*/*k *by using the nodes *t_j _*= *jh*, for *j *= 0, 1,..., *k*. Assume that $y\left(t\right),D_{*}^{\alpha }y\left(t\right),\text{and }D_{*}^{2\alpha }y\left(t\right)$ are continuous on [0, *a*] and use the generalized Taylor's formula (5) to expand *y*(*t*) about *t *= *t*_0 _= 0. For each value *t *there is a value *c*_1 _so that

(8) $$y\left(t\right)=y\left(t_{0}\right)+\left(D_{*}^{\alpha }y\left(t\right)\right)\left(t_{0}\right)\frac{t^{\alpha }}{\Gamma \left(\alpha +1\right)}+\left(D_{*}^{2\alpha }y\left(t\right)\right)\left(c_{1}\right)\frac{t^{2\alpha }}{\Gamma \left(2\alpha +1\right)}$$

When $\left(D_{*}^{\alpha }y\left(t\right)\right)\left(t_{0}\right)=f\left(t_{0},y\left(t_{0}\right)\right)$ and *h *= *t*_1 _are substituted into equation (8), the result is an expression for *y*(*t*_1_):

$$y\left(t_{1}\right)=y\left(t_{0}\right)+f\left(t_{0},y\left(t_{0}\right)\right)\frac{h^{\alpha }}{\Gamma \left(\alpha +1\right)}+\left(D_{*}^{2\alpha }y\left(t\right)\right)\left(c_{1}\right)\frac{h^{2\alpha }}{\Gamma \left(2\alpha +1\right)}$$

If the step size *h *is chosen small enough, then we may neglect the second-order term (involving *h*^2α^) and get

$$y\left(t_{1}\right)=y\left(t_{0}\right)+\frac{h^{\alpha }}{\Gamma \left(\alpha +1\right)}f\left(t_{0},y\left(t_{0}\right)\right)$$

The process is repeated and generates a sequence of points that approximates the solution *y*(*t*). The general formula for generalized Euler's method (GEM) when *t*_*j *+1 _= *t_j _*+ *h *is

(9) $$y\left(t_{j+1}\right)=y\left(t_{j}\right)+\frac{h^{\alpha }}{\Gamma \left(\alpha +1\right)}f\left(t_{j},y\left(t_{j}\right)\right)$$

for *j *= 0, 1,...,*k*-1. It is clear that if *α *= 1, then the generalized Euler's method (9) reduces to the classical Euler's method.

## 6. Numerical results

**Figure 1:**
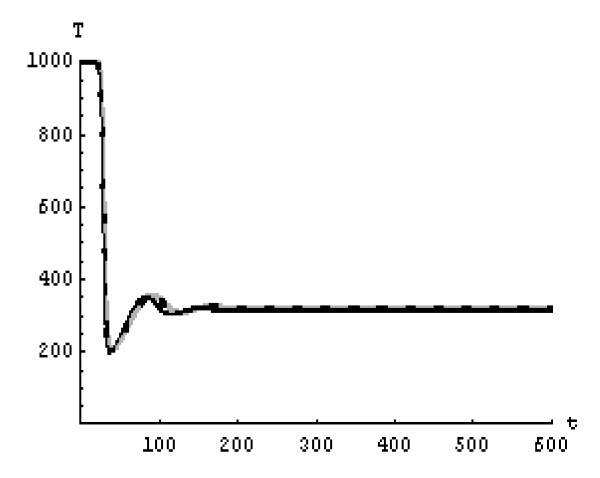
**The concentration of the uninfected CD4^+ ^T cells at *N *= 1000 in the 1^st ^case**. Gray solid line (α = 1), Dotted line (α = 0.99), Black solid line (α = 0.95).

**Figure 2:**
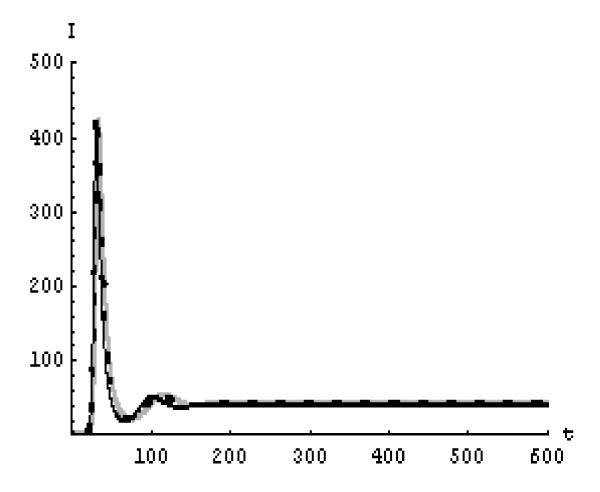
**The concentration of the infected CD4^+ ^T cells at *N *= 1000 in the 1^st ^case**. Gray solid line (α = 1), Dotted line (α = 0.99), Black solid line (α = 0.95).

**Figure 3:**
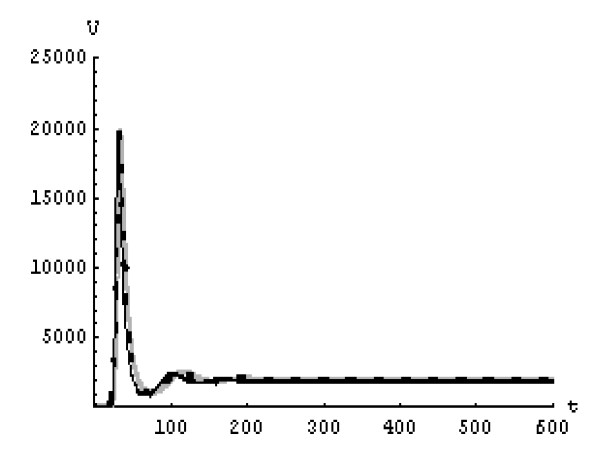
**The concentration of the free HIV virus particles at *N *= 1000 in the 1^st ^case**. Gray solid line (α = 1), Dotted line (α = 0.99), Black solid line (α = 0.95).

**Figure 4:**
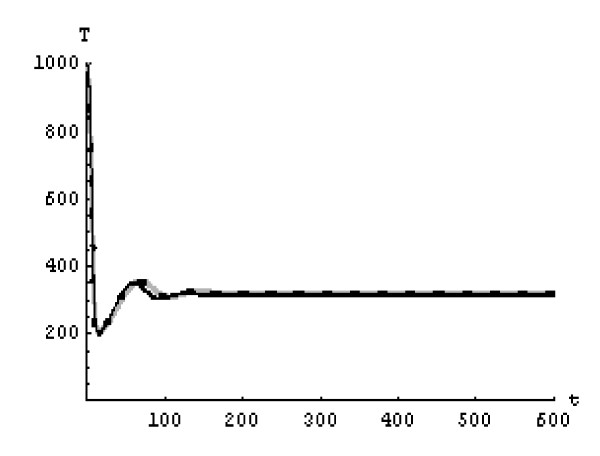
**The concentration of the uninfected CD4^+ ^T at *N *= 1000 in the 2^nd ^case**. Gray solid line (α = 1), Dotted line (α = 0.99), Black solid line (α = 0.95).

**Figure 5:**
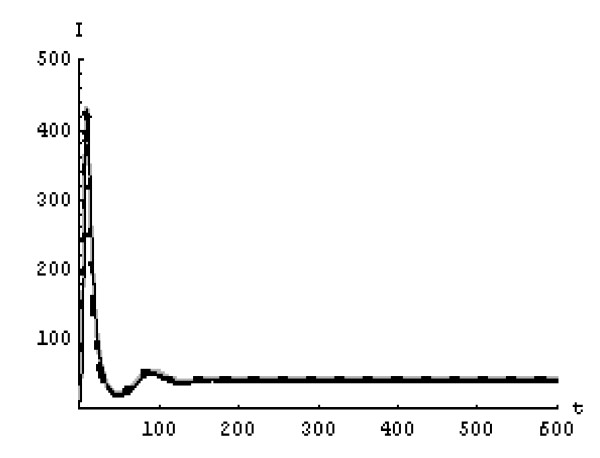
**The concentration of the infected CD4^+ ^T at *N *= 1000 in the 2^nd ^case**. Gray solid line (α = 1), Dotted line (α = 0.99), Black solid line (α = 0.95).

**Figure 6:**
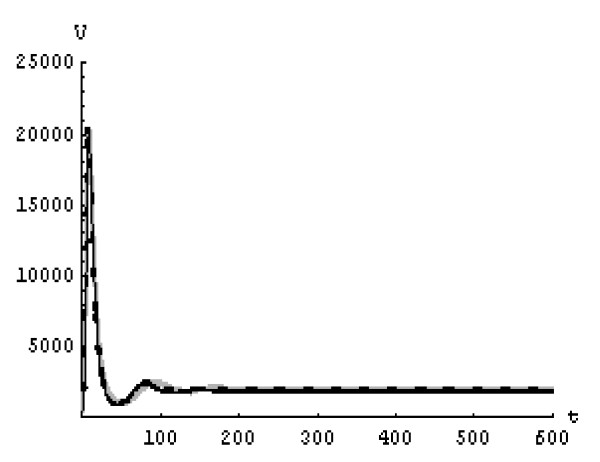
**The concentration of the free HIV virus particles at *N *= 1000 in the 2^nd ^case**. Gray solid line (α = 1), Dotted line (α = 0.99), Black solid line (α = 0.95).

**Figure 7:**
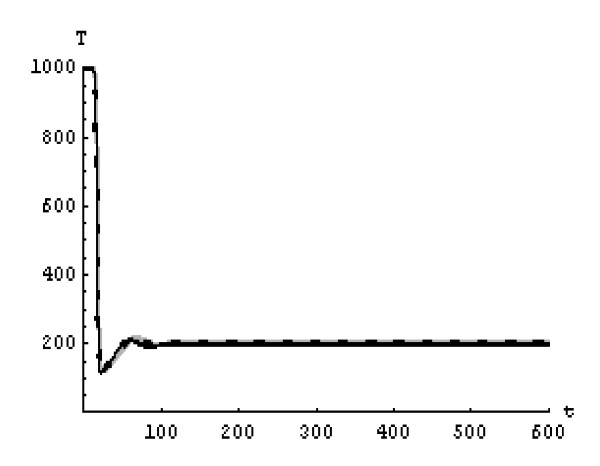
**The concentration of the infected CD4^+ ^T cells at *N *= 1600 in the 1^st ^case**. Gray solid line (α = 1), Dotted line (α = 0.99), Black solid line (α = 0.95).

**Figure 8:**
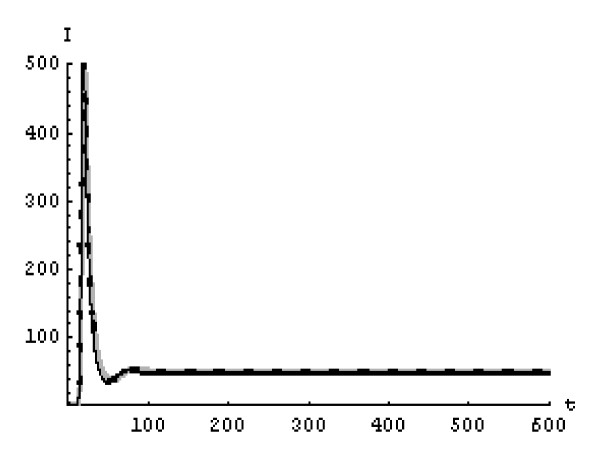
**The concentration of the infected CD4^+ ^T cells at *N *= 1600 in the 1^st ^case**. Gray solid line (α = 1), Dotted line (α = 0.99), Black solid line (α = 0.95).

**Figure 9:**
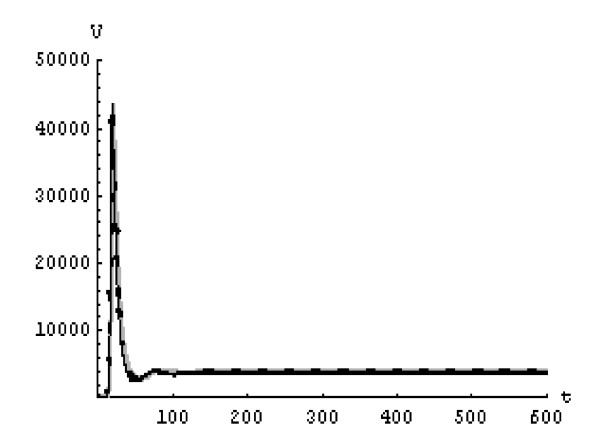
**The concentration of the free HIV virus particles at *N *= 1600 in the 1^st ^case**. Gray solid line (α = 1), Dotted line (α = 0.99), Black solid line (α = 0.95).

**Figure 10:**
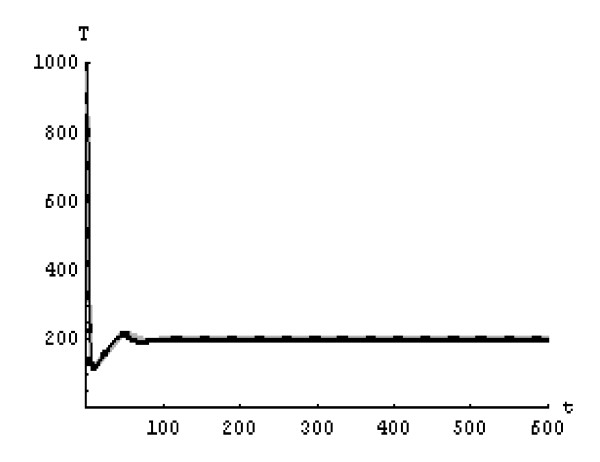
**The concentration of the uninfected CD4^+ ^T cells at *N *= 1600 in the 2^nd ^case**. Gray solid line (α = 1), Dotted line (α = 0.99), Black solid line (α = 0.95).

**Figure 11:**
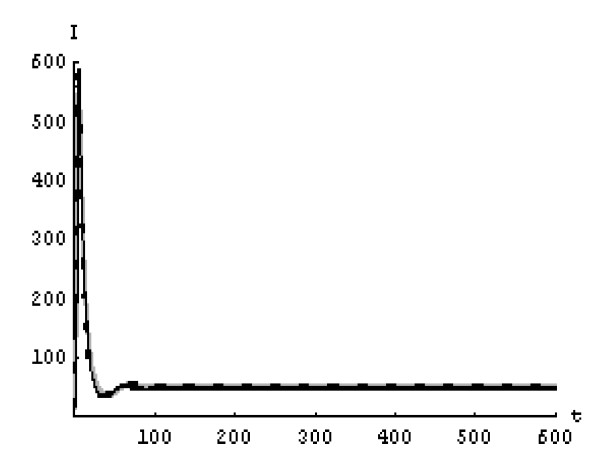
**The concentration of the infected CD4^+ ^T cells at *N *= 1600 in the 2^nd ^case**. Gray solid line (α = 1), Dotted line (α = 0.99), Black solid line (α = 0.95).

**Figure 12:**
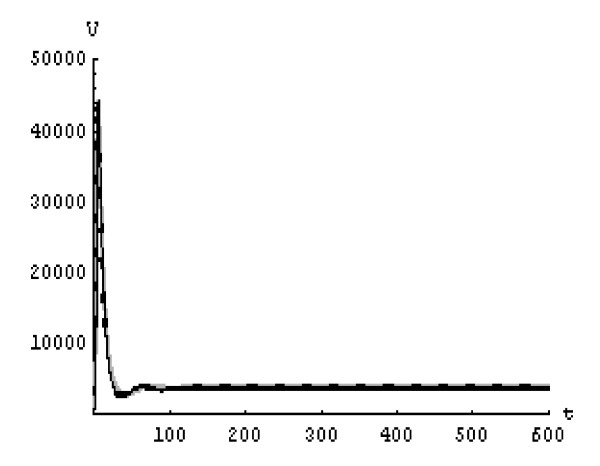
**The concentration of the free HIV virus particles at *N *= 1600 in the 2^nd ^case**. Gray solid line (α = 1), Dotted line (α = 0.99), Black solid line (α = 0.95).

We will solve the system (5) by using (GEM). Consider that *α*_1 _= *α*_2 _= *α*_3 _= *α*. We used the following data set: *s *= 10, *b *= 0.2, *k *= 0.000024, *d *= 0.01, *δ *= 0.16, *c *= 3.4, *N *varies. For this set of data *R*_0 _= 3.13 when *N *= 1000 (Figures 1, 2, 3, 4, 5, 6) and *R*_0 _= 5.01 when *N *= 1600 (Figures 7, 8, 9, 10, 11, 12). The initial conditions in the first case study are *T*(0) = 1000, *I*(0) = 0, *V*(0) = 0.001 while in the second case are *T*(0) = 1000, *I*(0) = 10, *V*(0) = 10. In the two cases the system goes to infected steady state.

## 7. Conclusion

In this paper we employed the Generalized Euler method (GEM) as a reasonable basis for studying the solution of human T-cell lymphotropic virus (HIV-I) infection of CD4^+ ^T-cells. We modified the integer-order model (1) into a fractional-order model (5). From the obtained results in the presented figures, it is clear that in the primary stage of the infection with the (HIV) virus, a dramatically decrease in the level of the CD4^+ ^T-cells occurs because of the death of such infected cells. On the other hand, the number of the free HIV virus particles and the number of susceptible CD4^+ ^T cells increase. This assumes that the growth of healthy T-cells slows down during the course of HIV infection. We have to give an attention to the parameter *b *which is called the reverting rate of infected cells to uninfected class due to non-completion of reverse transcription. Further, since only small fraction of infected cells will revert back due to incompletion of reverse transcription, we expect the reverting rate *b *to be small. The basic reproduction number of the presented model (5) is given in as:

$$R_{0}=\frac{N\delta Ks}{cd\left(b+\delta \right)}$$

It represents the average number of secondary infection caused by a single infected T cell in an entirely susceptible T cell population, throughout its infectious period. For system (5), if the basic reproduction number *R*_0 _≤ 1, the the virus is cleared and no HIV infection persists. If *R*_0 _> 1, the HIV infection persists in the T-cell population. In the two presented cases, *R*_0 _= 3.13 when *N *= 1000, (see Figures 1, 2, 3, 4, 5, 6) and *R*_0 _= 5.01 when *N *= 1600 (see Figures 7, 8, 9, 10, 11, 12), so the system goes to infected steady state. It is clear from the definition of *R*_0 _that *R*_0 _decreases as the reverting rate, *b *of infected cells increases, hence *R*_0 _can be low for a high parametric value of *b*. Increasing the *N *value will decrease the numbers of uninfected CD4^+ ^T-cells and increase the number of free virus substantially, but does not change the stability of the steady state. The concentration of susceptible CD4^+ ^T cells *T*(*t*), infected CD4^+ ^T cells *I*(*t*), and free HIV virus particles *V*(*t*) in the blood have been obtained, therefore when *α *→ 1 the solution of the fractional model (5) *T*_α _(*t*), *I*_α_(*t*), *V*_α_(*t*), reduce to the standard solution *T*(*t*), *I*(*t*), *V*(*t*). Finally, the recent appearance of fractional differential equations as models in some fields of applied mathematics makes it necessary to investigate methods of solution for such equations (analytical and numerical) and we hope that this work is a step in this direction.

## Competing interests

The authors declare that they have no competing interests.

## Authors' contributions

AA projected and coordinated the numerical experiments. SR participated the main computer programming. MK performed the numerical simulation. All authors read and approved the manuscript.
